# Increased body mass and depressive symptomatology are associated with hypercholesterolemia, among elderly individuals; results from the MEDIS study

**DOI:** 10.1186/1476-511X-8-10

**Published:** 2009-03-30

**Authors:** Stefanos Tyrovolas, Christos Lionis, Akis Zeimbekis, Vassiliki Bountziouka, Mary Micheli, Alexia Katsarou, Natassa Papairakleous, George Metallinos, Kornilia Makri, Evangelos Polychronopoulos, Demosthenes B Panagiotakos

**Affiliations:** 1 Department of Nutrition Science – Dietetics, Harokopio University, Athens, Greece; 2 School of Medicine, University of Crete, Heraklion, Greece; 3 Health Center of Kalloni, General Hospital of Mitilini, Mitilini, Greece

## Abstract

**Background:**

Hypercholesterolemia is one of the most important factors causing cardiovascular disease (CVD). The aim of the present work was to evaluate the relationships between socio-demographic, clinical, lifestyle and depression status and the presence of hypercholesterolemia, among elderly individuals without known CVD.

**Methods:**

During 2005–2007, 1190 elderly (aged 65 to 100 years) men and women (from Cyprus, Mitilini, Samothraki, Cephalonia, Crete, Lemnos, Corfu and Zakynthos) were enrolled. Socio-demographic, clinical and lifestyle factors were assessed through standard procedures. Symptoms of depression were evaluated using the short-form of the Geriatric Depression Scale (GDS, range 0–15). Dietary habits were assessed through a semi-quantitative food frequency questionnaire. Hypercholesterolemia was defined as total serum cholesterol > 200 mg/dL or use of lipids lowering medication.

**Results:**

44.6% of males and 61.9% of females had hypercholesterolemia (p < 0.001). Only, 63% of hypercholesterolemic participants were under special diet or pharmaceutical treatment. Hypercholisterolemic individuals had higher prevalence of obesity (43% vs. 25%), hypertension (76% vs. 57%) and diabetes (25% vs. 17%) compared with normal participants (p < 0.001). Furthermore, hypercholisterolemic participants showed higher depression levels (p = 0.002). After adjusting for various confounders, GDS score and BMI correlated with 13% (95%CI 0.98–1.30) and 14% (95%CI 0.99–1.31) higher likelihood of having hypercholesterolemia.

**Conclusion:**

A considerable proportion of our elderly sample had hypercholesterolemia, while 1/3 of them were untreated. Furthermore, presence of hypercholesterolemia was correlated with depressive symptomatology and increased BMI.

## Introduction

Current epidemiologic data suggest that hypercholesterolemia is the main contributor for atherogenesis [1-3]. Recent guidelines recommend that it is important to maintain low cholesterol levels in order to reduce mortality from cardiovascular disease [4]. Despite these considerations, the management of blood lipids levels is difficult, especially in older adults. Several studies have suggested that hypercholesterolemia is associated with lifestyle habits, like dietary, smoking and physical inactivity status. Moreover, the effect of abnormal blood lipids levels on cardiovascular system is enhanced with increased age. Taking into account that between the years 2000 and 2050 the world wide proportion of persons over 65 years of age is expected to increase from the 6.9% to 16.4% [5], the burden of hypercholesterolemia in the future is also expected to increase at alarming rates. Therefore, taking into account the growing population of elderly in industrialized countries the investigation of associations between risk factors and life-style, and psycho-social factors is of major interest. Specifically, various anthropometric indices including body mass index (BMI), waist and hip circumferences and waist-to-hip ratio, have been employed to capture the obesity-related cardiovascular risk. Moreover, several studies have consistently shown that both absolute total fat and adipose tissue distribution are strongly associated with the risks of hyperlipidemia as well as diabetes and hypertension [4]. As for the psychological factors, depression is one of them influencing many people around the world, through lifespan. It is now evident from many studies that depression affects approximately 121 million people worldwide, while higher rates are often observed among the elderly [6]. Depressive symptoms have been associated with increased cardiovascular mortality [7,8] and diabetes in the elderly men [7,9] and, in some studies, with increased total cholesterol levels [10,11]. However, the role of depression on lipid disorders has rarely been investigated, especially in elderly people living in the Mediterranean region, where the protective effect of diet may influence the aforementioned relationship.

Therefore, since data regarding the epidemiology of hypercholesterolemia among elderly people are sparse in the literature, the aim of the present work was to evaluate the relationship between various socio-demographic, clinical, anthropometric, lifestyle and psychological characteristics with the presence of hypercholesterolemia, among men and women older than 65 years, living in the Mediterranean islands.

## Methods

### Participants

The MEDIS study [12] is a health and nutrition survey that aimed to evaluate bio-clinical, lifestyle, behavioural and dietary characteristics of 1190 elderly people living in Mediterranean islands. All participants were without any clinical evidence of CVD or cancer in their medical history. A random, population-based, multistage sampling method (i.e., age group, 3 levels (65–75, 75–85, 85 ±) and 2 sex levels) was used to select men (76 ± 7 years) and women (74 ± 7 years), from Cyprus Republic and Mitilini, Samothraki, Cephalonia, Crete, Lemnos, Corfu and Zakynthos islands, in Greece. Individuals residing in assisted-living centres, as well as those with a clinical history of CVD were not included in the survey. The target sample size was 300 people from Cyprus, 150 from each one of the other islands. Of the initially selected people, 553 men and 637 women (n = 1190) agreed to participate (Cyprus, n = 300, Mitilini, n = 142, Samothraki, n = 100, Cephalonia, n = 114, Crete, n = 131, Corfu, n = 150, Lemnos n = 150, Zakinthos, n = 103). Of them, 460 (39%) were living in rural areas of the islands. The participation rate varied from 75% to 89%. A group of health scientists (i.e., physicians, dieticians and nurses) with previous experience in field investigation collected all the required information, using a quantitative questionnaire and standard procedures.

The retrieved data were confidential, and the study followed the ethical consideration that provided by the World Medical Association (52^nd ^WMA General Assembly, Edinburgh, Scotland, October 2000). Before the interview, participants were informed about the aims and procedures of the study, and signed an informed consent.

### Measurements

The retrieved information included basic demographic characteristics, such as age, gender, annual income, lifestyle factors, as well as various bio-clinical characteristics. Particularly, current smokers were defined as those who smoke at least one cigarette per day or have stopped cigarette smoking during the past 12 months. Former smokers were defined as those who previously smoked, but have not done so in a year or more. The remaining participants were defined as rare- or non-smokers. Exposure to environmental tobacco smoke (at workplace, home or restaurants, etc.) for more than 30 minutes per day assisted us to define people as passive smokers. Dietary habits were assessed through a semi-quantitative, validated and repeatable food-frequency questionnaire. Frequency of consumption of various food groups and beverages (i.e., meat and products, fish and fisheries, milk and other dairy, fruits, vegetables, greens and salads, legumes, cereals, coffee and tea and soft-drinks) on daily, weekly or monthly basis, was assessed. Furthermore, intake of various alcoholic beverages (i.e., wine, beer, etc.) was measured in terms of wineglasses adjusted for ethanol intake (e.g., one 100 ml glass of wine was considered to have 12% ethanol). To better evaluate overall dietary habits the MedDietScore (range 0–55) was used in order to assess the level of adherence to this traditional dietary pattern [13]. Higher values of this diet score indicates greater adherence to the Mediterranean diet. Physical activity was evaluated using the shortened version of the self-reported, International Physical Activity Questionnaire (IPAQ) for the elderly [14]. Frequency (times per week), duration (minutes per time) and intensity of physical activity during sports, occupation and/or free-time activities were assessed. Participants were classified as inactive, minimally active and HEPA active (health enhancing physical activity; a high active category), based on the following criteria: inactive was classified an individual when no criteria were met to classify him or her in any of the other two categories; minimally active, which is the classification for sufficiently active, when any of the following three criteria were met: a) 3 or more days of vigorous activity of at least 20 minutes per day, b) 5 or more days of moderate-intensity activity or walking of at least 30 minutes per day, or c) 5 or more days of any combination of walking, moderate-intensity or vigorous-intensity activities achieving of at least 600 MET-min/week; and HEPA active when any of the following criteria were met: a) vigorous-intensity activity on at least 3 days achieving a minimum of at least 1500 MET-minutes/week, or b) 7 or more days of any combination of walking, moderate-intensity or vigorous intensity activity achieving a minimum of at least 3000 MET-minutes/week. Participants were instructed to report only episodes of activities of at least 10 minutes, since this is the minimum required to achieve health benefit.

Furthermore, diabetes mellitus (type 2) was determined by fasting plasma glucose tests and was analyzed in accordance with the American Diabetes Association diagnostic criteria (i.e., fasting blood glucose levels greater than 125 mg/dl or use of special medication, indicated the presence of diabetes). Moreover, participants' who had blood pressure levels ≥ 140/90 mmHg or used antihypertensive medications were classified as hypertensive. Fasting blood lipids levels were also recorded and hypercholesterolemia was defined as total serum cholesterol levels > 200 mg/dl or the use of lipid-lowering agents. HDL-, LDL-cholesterol and triglycerides were also recorded. Weight and height were measured to attain body mass index (BMI) scores (kg/m^2^). Obesity was defined as BMI > 29.9 Kg/m^2 ^[15].

### Assessment of depressive symptoms

Symptoms of depression during the past month were assessed using the validated Greek translation of the shortened, self-report, Geriatric Depression Scale (GDS) [16-18]. The following 'yes or no' items were included in the GDS questionnaire: "*Are you basically satisfied with your life? Have you dropped many of your activities and interests? Do you feel that your life is empty? Do you often get bored? Are you in good spirits most of the time? Are you afraid that something bad is going to happen to you? Do you feel happy most of the time? Do you often feel helpless? Do you prefer to stay at home, rather than going out and doing new things? Do you feel you have more problems with memory than most? Do you think it is wonderful to be alive now? Do you feel pretty worthless the way you are now? Do you feel full of energy? Do you feel that your situation is hopeless? Do you think that most people are better off than you are?*" Responses were coded with 1s (for positive answers) and 0s (for negative answers) yielding a total possible score between 0 and 15. For clinical purposes, GDS scores have been used to indicate no depression (0 – 4), mild depression (5 – 10) or severe depression (11 – 15) [16]. This same classification system will be used in many of the subsequent analyses. In order to increase the precision of the retrieved psychological information from the participants, we asked a close friend, companion, or sibling of them to answer the same questions regarding the participant's psychological status. Both data sets (i.e., from participants and their friends, companion or siblings) were compared using Kendall's-τ coefficient. Participants with significant discordance (i.e., > 3%) from their counterparts were excluded from the psychological analyses (4 participants were excluded).

### Statistical analyses

Continuous variables are presented as mean values ± standard deviation. The categorical variables are presented as frequencies. After controlling for equality of variances (homoscedacity), associations between continuous variables and group of participants are evaluated with analyses of variance (ANOVA). To correct for the inflation of Type-I error in multiple comparisons, Bonferroni's correction is applied. Associations between continuous variables (e.g., GDS score and total serum cholesterol levels) are tested with Spearman's correlation coefficient. Stepwise multiple logistic regression analysis evaluated the effect of various characteristics of the participants on the likelihood of having hypercholesterolemia, after adjusting for various potential confounders. Deviance residuals were calculated to evaluate model's goodness-of-fit. P-values < 0.05 from two-sided hypotheses are considered as statistically significant. All statistical calculations are performed on the SPSS version 14.0 software (SPSS Inc, Chicago, IL, U.S.A.).

## Results

We observed that 44.6% of males and 61.9% of females had hypercholesterolemia (p < 0.001). Only 63% of hypercholesterolemic participants were under special diet or pharmaceutical treatment. Moreover, 22% of the participants reported that they rarely controled their blood lipids levels, while 48% of the participants check their blood lipids once every 6 months. The rest never control their lipid profile. No differences were observed between genders. Regarding the other characteristics, 69% of the participants showed mild to severe levels of depression (i.e., mean GDS score 8.2 ± 4.3).

Table 1 presents the characteristics of the participants, by cholesterol levels. As we can observe, that participants with hypercholesterolemia appeared a significantly higher level of BMI (*p *< 0.001). It also seems that participants with high cholesterol levels had significantly lower percentages of cigarette smoking than elderly with no hypercholesterolemia (*p *< 0.001). Furthermore, in elderly with high blood cholesterol levels the prevalence of hypertension, obesity and diabetes was significantly higher than in participants with no hypercholesterolemia (*p *< 0.001). Participants with hypercholesterolemia presented significantly higher values of diastolic blood pressure (*p *= 0.039) than the group of the elderly with normal cholesterol levels. Also, the group with hypercholesterolemia seems to have higher levels of serum triglycerides than the group with normal levels of blood cholesterol (*p *< 0.001). Regarding dietary habits, the mean diet score suggests that the level of adherence to the Mediterranean diet was 61.2% for males (score 33.7 × 100/55) and 60.6% for females (score 33.3 × 100/55), while adherence to the Mediterranean diet was not associated with the presence of hypercholesterolemia (moderate vs. low adherence: odds ratio = 0.87, 95% CI 0.65 to 1.17, high vs. low adherence: odds ratio = 0.90, 95% CI 0.68 to 1.19), after adjusting for age and gender. However, elderly people with hypercholesterolemia had lower percentage in the consumption of alcohol than the people with high levels of blood cholesterol (*p *< 0.001). Finally, participants with hypercholesterolemia showed significantly higher levels of depression than the elderly with normal cholesterol levels (*p *= 0.002). Age-gender adjusted analysis showed a positive association between GDS score and the likelihood of having hypercholesterolemia (95% CI 1.01 to 1.10).

**Table 1:** Socio-demographic, clinical and lifestyle characteristics of the participants by cholesterol levels.

	**Normal cholesterol levels** **(*n *= 536)**	**Hypercholesterolemia** **(*n *= 625)**	***p***
Age (years)	74.6 ± 7.6	73.4 ± 6.2	0.004
Male gender (%)	56	39	< 0.001
Body Mass Index (kg/m^2^)	27.6 ± 4.5	29.5 ± 4.9	< 0.001
Education level (yrs. of school)	6.02 ± 3.4	5.76 ± 3.0	0.183
Physical activity (%)	37.5	34.5	0.290
Current or former smoker (%)	19	10	< 0.001
MedDietScore (0–55)	33.6 ± 4.0	33.5 ± 4.0	0.812
Alcohol (> 6 gr ethanol/day,%)	46	33	< 0.001
Hypertension (%)	57	76	< 0.001
Obesity (%)	24.8	42.9	< 0.001
Diabetes (%)	17	25	< 0.001
Geriatric Depression Scale (0–15)	7.6 ± 4.4	8.7 ± 4.2	0.002
Triglycerides (mg/dL)	112.4 ± 41.2	141.7 ± 67.7	< 0.001
Fasting glucose (mg/dL)	115.8 ± 41.1	116.3 ± 41.5	0.884
Systolic blood pressure (mmHg)	139.1 ± 17.6	139.1 ± 17.1	0.979
Diastolic blood pressure (mmHg)	78.7 ± 9.4	79.9 ± 9.4	0.039

Table 2 presents the factors which affect the presence of hypercholesterolemia in the participants. Particularly after adjusting for age, gender, BMI, MedDietScore, GDS, hypertension, diabetes, education level, physical activity and smoking, it seems that 1 year difference in age per 1 was associated with 10% lower of hypercholesterolemia (95% CI 0.81 to 1.01). Additionally, increase of BMI per 1 kg/m^2 ^is associated with 13% higher likelihood of having hypercholesterolemia (95% CI 0.98 to 1.30). Finally, 1 unit increase in the GDS score (i.e., roughly 1%), is associated with 14% higher likelihood of having hypercholesterolemia (95% CI 0.99 to 1.31).

**Table 2:** Results from stepwise multiple logistic regression

**Initial model**		
	**Odds ratio**	**95% confidence interval**
Age (per 1 year)	0.89	0.77–1.02
Males vs. females	0.44	0.06–2.86
Body Mass Index (per 1 kg/m^2^)	1.10	0.92–1.32
Financial status (high vs. low)	0.60	0.24–1.45
MedDietScore (per 1 unit)	1.02	0.85–1.21
GDS (per 1 unit)	1.10	0.94–1.29
Hypertensive (y/n)	0.86	0.17–4.29
Diabetes (y/n)	1.08	0.15–7.64
Education level (per 1 year of school)	0.97	0.76–1.25
Physical activity (y/n)	0.54	0.11–2.61
Smoking (y/n)	0.97	0.91–1.03
**Final model**		
Age (per 1 year)	0.90	0.81–1.01
Body Mass Index (per 1 kg/m^2^)	1.13	0.98–1.30
GDS (per 1 unit)	1.14	0.99–1.31

## Discussion

This epidemiological survey evaluated factors associated with the presence of hypercholesterolemia among elderly people living in Mediterranean islands. Data analysis showed that a considerable proportion of the participants (over 40% of males and females) had hypercholesterolemia, while 4 out of 10 of them were untreated. Moreover, depression status and increased BMI were independently correlated with the presence of hypercholesterolemia.

There are very few epidemiological studies that have assessed blood lipids levels among elderly people, in Greece or other Mediterranean areas. For example, the ATTICA epidemiological study [19] showed that 49% of men and 52% of women, aged > 50 years, had high total cholesterol levels (i.e. > 200 mg/dl). Furthermore, from the ATTICA study, the five-year incidence of hypercholesterolemia in men and women > 50 years old was close to 40% for males and 60% for females [20]. The cardio-epidemiological study presented that the prevalence of hypercholesterolemia, in males and females aged > 60 years, healthy or with acute coronary syndrome were 60% for males and close to 30% for the females [21]. Another epidemiological study showed that approximately 30% of males over 55 years old and approximately 45% of females, had high total cholesterol levels (i.e. > 200 mg/dl) [22]. At another Mediterranean area, Costa et al. [23] reported that the prevalence of total cholesterol levels > 200 mg/dl among elderly people was 57%. Also, the MEDIS study showed for the elderly of Cyprus, that sixty five percent of the participants had hypercholesterolemia (60% of men and 68% of women). In addition to this, 16% of men and 42% of women had cholesterol levels above 240 mg/dl [24]. Moreover, according to the National Health and Nutrition Examination Survey (NHANES) III in USA, roughly 50% of white adult men and women in the USA had total blood cholesterol levels of over 200 mg/dl, [25]. Furthermore, several studies have shown that a higher percentage of women than men have total blood cholesterol of 200 mg/dl or higher, beginning at age 50 [26,27,2]. The later was confirmed by our study, too.

Unadjusted data analysis revealed that the presence of hypertension, diabetes and the levels of serum triglycerides were higher in the elderly with high blood cholesterol levels. Moreover, the consumption of alcohol seems to decrease significantly as blood cholesterol levels increase, on the other hand the level of adherence to the Mediterranean diet did not show any difference between normal and hypercholesterolemic participants. However, all the aforementioned associations lost there significance when various confounders were taken into account. Further multi-adjusted data analysis, revealed a positive correlation between BMI and presence of hypercholesterolemia. This finding seems to agree with the results from other studies that reported also the same correlation [28-31]. In these surveys, increased BMI is associated with increased total serum cholesterol, LDL-cholesterol and triglycerides. The previous is not something new since lipid metabolism is closely connected to the metabolism of carbohydrates which may be converted to fats, and, consequently to increased body mass. It is well known that BMI, where there is excess abdominal fat, is correlated with insulin resistance [32,33]. Insulin resistance also results in the decreased cleaning of FFAs from the plasma. Therefore, the lipidaimic profile of someone with increased BMI shows increased levels of VLDL-TG LDL-CHOL, TG. [20,34]. Insulin resistance is also linked to high cholesterol synthesis and decreased cholesterol absorption [35]. On the other hand, a diet high in trans and saturated fatty acids increases blood cholesterol and LDL-cholesterol levels [36], where a diet rich in carbohydrates increases blood triglycerides [37]. All these and the fact that our sample presented a modest adaptation to the Mediterranean diet, we may explain why there is a strong correlation between BMI and hypercholesterolemia in our sample. On the other hand, no correlation was found between hypercholesterolemia and Mediterranean diet. Benefits from the Mediterranean dietary pattern on blood lipids have rarely been reported in the literature, especially in the elderly. In our sample the absence in correlation between hypercholesterolemia and the Mediterranean diet may have come up because of the modest Mediterranean diet score that our sample presented (60%). In general, elderly people showed problems in their nutrition due to health problems [38] as well as due to their low annual income, which discourages them to consume a global diet with food from all the food groups. A higher diet score could possibly enhance the presence of such a correlation.

We observed a strong correlation between depression status and presence of hypercholesterolemia, which has been presented from some other studies, too [10,11]. Evidence suggests that depression, even in the elderly, has both behavioral and pathophysiological effects on health and especially on cardiovascular system. Firstly, depression has been associated with several unhealthy lifestyle behaviors, such as poor adherence to medical regimens, smoking, unhealthy dietary habits and physical inactivity [39,40], which may increase the risk of hypercholesterolemia. Additionally, pathophysiological effects suggest that multiple bio-clinical factors, such as genetic, biochemical (inflammation and coagulation markers, arterial blood pressure, heart rate variability etc.) and psychodynamic (through the increment in vascular risk), may interact with depression status in complex ways, promoting the development of hypercholesterolemia, CVD and other chronic diseases [41].

### Strengths and Limitations

The present study has several strengths since it is one of the first studies that evaluated a large sample of "healthy", free-living elderly people living in the Mediterranean islands. Moreover, these people belong to the generation of individuals that the traditional Mediterranean type of diet was firstly originated. Therefore, the evaluation of health status of these people, and the assessment of factors that may have influenced it, deserves special attention. However, this study shares some limitations mainly because of its cross-sectional design. Thus, the findings may be influenced by potential recall bias, particularly in the assessment of depression and dietary habits.

## Conclusion

Considering the growing population of elderly patients in industrialized nations the investigation of associations between cardiovascular risk factors and life-style, social as well as psychological factors among elderly patients surely is of interest. As this is grossly influenced by national and cultural factors, different regional studies are necessary to elucidate this topic. In the present work a large proportion of the investigated sample had high cholesterol levels, while roughly 4 out of 10 of them were untreated. Depression and body mass were associated with the presence of hypercholesterolemia, even after adjusting for various covariates. Taking into account the increased depression rates observed in the elderly, co-existence of this psychological disorder with high blood lipids levels may promote the development of cardiovascular disease.

## Competing interests

The authors declare that they have no competing interests.

## Authors' contributions

ST: wrote the paper, performed the data analyses and participated in the enrolment of the participants, CL, AZ, EP: supervised the study and reviewed the paper, VB, MM, AK, NP, GM, KM: participated in the field investigation and DP: supervised the data analyses, wrote the paper and designed and coordinated the study.
